# Calcification Microstructure Reflects Breast Tissue Microenvironment

**DOI:** 10.1007/s10911-019-09441-3

**Published:** 2019-12-05

**Authors:** Sarah Gosling, Robert Scott, Charlene Greenwood, Pascaline Bouzy, Jayakrupakar Nallala, Iain D. Lyburn, Nicholas Stone, Keith Rogers

**Affiliations:** 1 Cranfield Forensic Institute, Cranfield University, Shrivenham, UK; 2 School of Chemical and Physical Sciences, Keele University, Keele, Staffordshire UK; 3 School of Physics and Astronomy, University of Exeter, Exeter, UK; 4 Thirlestaine Breast Centre, Gloucestershire Hospitals NHS Foundation Trust, Cheltenham, Gloucestershire UK

**Keywords:** Hydroxyapatite, Carbonate, Breast Cancer, Calcification, X-ray diffraction

## Abstract

Microcalcifications are important diagnostic indicators of disease in breast tissue. Tissue microenvironments differ in many aspects between normal and cancerous cells, notably extracellular pH and glycolytic respiration. Hydroxyapatite microcalcification microstructure is also found to differ between tissue pathologies, including differential ion substitutions and the presence of additional crystallographic phases. Distinguishing between tissue pathologies at an early stage is essential to improve patient experience and diagnostic accuracy, leading to better disease outcome. This study explores the hypothesis that microenvironment features may become immortalised within calcification crystallite characteristics thus becoming indicators of tissue pathology. In total, 55 breast calcifications incorporating 3 tissue pathologies (benign – B2, ductal carcinoma in-situ - B5a and invasive malignancy - B5b) from archive formalin-fixed paraffin-embedded core needle breast biopsies were analysed using X-ray diffraction. Crystallite size and strain were determined from 548 diffractograms using Williamson-Hall analysis. There was an increased crystallinity of hydroxyapatite with tissue malignancy compared to benign tissue. Coherence length was significantly correlated with pathology grade in all basis crystallographic directions (*P* < 0.01), with a greater difference between benign and in situ disease compared to in-situ disease and invasive malignancy. Crystallite size and non-uniform strain contributed to peak broadening in all three pathologies. Furthermore, crystallite size and non-uniform strain normal to the basal planes increased significantly with malignancy (*P* < 0.05). Our findings support the view that tissue microenvironments can influence differing formation mechanisms of hydroxyapatite through acidic precursors, leading to differential substitution of carbonate into the hydroxide and phosphate sites, causing significant changes in crystallite size and non-uniform strain.

## Introduction

Breast cancer is the most commonly diagnosed cancer in women in the UK [1]. Screening mammography potentially identifies cases at an early stage by analysing features, including the presence of masses, asymmetric soft tissue densities and microcalcifications [2, 3]. Microcalcifications are deposits of calcium salts with diameters not exceeding 1 mm [4]. Mammographic interpretation is based on distribution, size and morphology and may result in patients being recalled for further investigation through core needle biopsy. However, only 21% of women recalled for biopsy receive a subsequent diagnosis of cancer, meaning that the positive predictive value of mammography needs to be improved upon [2]. Studies have shown malignant calcifications identified through mammography are mostly related to pure DCIS (64%) or DCIS with invasive foci (32%) but rarely with pure invasive carcinoma (4%) [5]. Therefore, mammographically identified calcifications are predominantly used for discriminating cases of DCIS from benign, and between DCIS grades.

The calcification based on chemical composition may be broadly split into two categories; type I are amber, partially translucent and birefringent, whereas type II, are grey-white and opaque [6]. Type I calcifications consist of calcium oxalate dihydrate (CaC_2_O_4_.2(H_2_O)) and are predominantly found associated with benign conditions, whereas type II calcifications are composed primarily of calcium phosphate in the form of hydroxyapatite (Ca_10_(PO_4_)_6_(OH)_2_) and are associated with benign, in-situ and cancerous breast tissues [6]. These apatite calcifications consist of crystallites with nanometre dimensions.

To date, numerous in vivo formation mechanisms for hydroxyapatite (HAp) calcification have been postulated. Evidence of osteoblast-like cells has been observed in breast cancer, which may implicate a bone-like deposition mechanism in cancer cells [7]. There is also evidence for an imbalance of mineralisation promoters and inhibitors in breast cancer, such as alkaline phosphatase which may cause calcification [8, 9]. This mechanism may be linked to a secretory process where secreted vesicles are calcified in the extracellular matrix [10]. Additionally, necrosis is a common feature of high-grade DCIS, which may act as a nucleation centre for calcifications [10]. While there is evidence to support these mechanisms, the actual process of HAp formation from precursors in breast tissue is relatively unexplored. The mechanism of HAp deposition can occur through a number of precursors such as amorphous calcium phosphate (ACP) (Ca_9_(PO_4_)_6_), and often other intermediate phases [11]. Such precursor phases are dependent on factors such as environmental pH and temperature, which can affect the kinetics of ACP to HAp transformation, as well as the progression through other intermediate phases. For example, at low pH, acidic precursors such as octacalcium phosphate (OCP) (Ca_8_(HPO_4_)_2_(PO_4_)_6_·5H_2_O) and dicalcium phosphate dihydrate (DCPD) are more likely to form prior to HAp formation, whereas at neutral or alkaline pH, evidence suggests the more direct conversion of ACP to HAp [11].

Cancerous tissues are known to have a wide array of differences to normal and benign tissues in terms of their microenvironments, with some of these differences also evident between ductal carcinoma in-situ (DCIS – herein referred to as ‘in-situ*’*) and invasive cancer cells. This includes gene and protein expression, such as oestrogen and progesterone receptor positivity; inflammation; immune cells recruitment such as leukocytes and ion trafficking [12, 13]. Some factors affecting the formation mechanisms of HAp also differ between the microenvironments of cancerous and normal breast tissues. For example, the extracellular pH of invasive cancer cells is more acidic than benign and in-situ cells, hence an increased cell invasiveness [12]. It is the combination of these changes that permits the initial carcinogenesis and the progression from in-situ to invasive and metastatic cancers by maintaining feedback loops, enabling angiogenesis and facilitating cellular migration [13]. While histopathology of biopsies can analyse some of these characteristic features, how these environments influence mammographic findings including microcalcifications has not previously been explored.

**Fig. 1:**
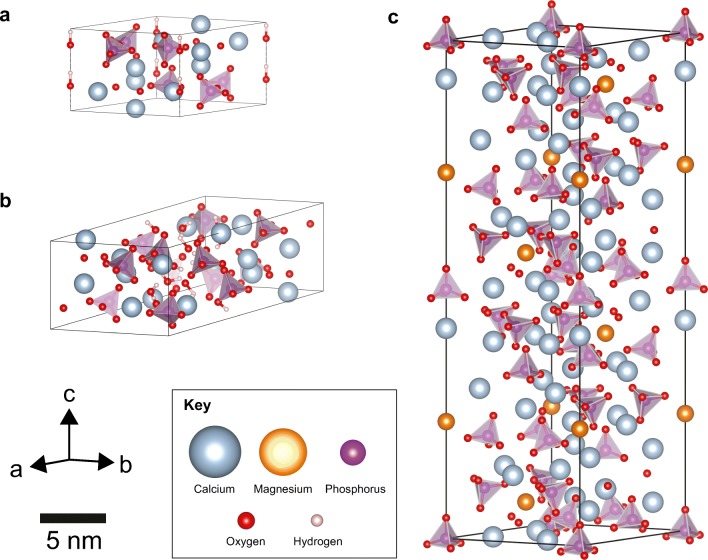
The structure of single repeating units (unit cells) of breast microcalcification phases. **a** a unit cell of hydroxyapatite (HAp), **b** a unit cell of octacalcium phosphate (OCP), **c** a unit cell of magnesium whitlockite. Arrows indicate the three directions of the lattice parameters ‘a’, ‘b’ and ‘c’

Physicochemical differences between HAp microcalcifications associated with benign proliferative conditions, in-situ carcinoma and invasive cancers have been observed by previous studies [14, 15]. For example, sodium incorporation in the HAp has been reported to increase with tissue malignancy [15]. Further, the amount of β-tricalcium phosphate (whitlockite) as the magnesium substituted phase within type II calcifications has been shown to quantitatively increase with increasingly malignant tissue [15]. The structures of HAp, OCP and whitlockite are shown in Fig. 1.

HAp is an enigmatic material, that can accommodate multiple substitutional ions, which affects the crystallographic properties determined using X-ray diffraction (XRD) techniques [16–18]. Therefore, XRD is a useful tool in determining fine structural differences in type II breast calcifications. XRD is used to investigate multiple properties, including ‘crystallinity’, ‘coherence length’, ‘lattice parameters’, crystallite size and non-uniform strain. Here, crystallinity refers to the degree of order found within the crystal structure, with the less crystalline materials exhibiting greater diffraction peak broadening [18]. Coherence length is the average (direction dependent) distance within a crystal that lattice order persists. Decreasing coherence length increases the magnitude of peak broadening therefore can be used as a quantitative measure of crystallinity [19]. Lattice disorder arises from a number of phenomena and coherence length is therefore often separated into crystallite size, (the average physical dimension individual crystallites in a given direction), and non-uniform, or inhomogeneous, strain (intra-crystallite imperfections) [19]. Finally, lattice parameters define the shape and lengths of the individual, molecular repeating units (unit cells) that make up the crystallite structure. These are denoted as ‘a’ and ‘c’ for HAp [18].

Numerous substitutions into the HAp lattice have been observed. For example, halide ions can substitute for hydroxide, hydrogen phosphate substitutes into the phosphate site and sodium and magnesium can substitute for calcium [16]. Perhaps most significantly, carbonate ions have been shown to substitute into the HAp lattice in two distinct sites: at the hydroxyl site (A-type); and the phosphate site (B-type). Additionally, carbonate can adsorb onto the surface of HAp crystals, where it is labile and does not form part of the lattice [18]. In synthetic preparations of hydroxyapatite, the type of carbonate substitution has been shown to be affected by the carbonate concentration in the surrounding environment, with higher carbonate concentrations, up to 4 wt%, favouring A-type carbonate and lower carbonate concentrations favouring B-type carbonate, though the opposite trend is observed beyond 4 wt% [20]. Carbonate substitution is also known to have several effects on the properties of HAp, for example, increasing carbonate substitution has been shown to increase HAp solubility [21].

It is generally accepted that all biological apatites contain carbonate substitution to some extent, varying from 2 to 6% weight in enamel, dentine and bone, and is thought to be dominated by B-type substitution, with A/B type ratio ranging from 0.7–0.9 [18, 22–24]. In synthetic apatites, increasing total levels of carbonate substitution have been correlated with decreasing crystallite size, and increasing non-uniform strain [17, 21, 25]. Previous studies have also demonstrated a decrease in total carbonate concentration in calcifications with increasing tissue malignancy, from benign to invasive, in the range of 1–2 wt% [22, 26]. Together, an increase in crystallite size and decrease in non-uniform strain with increasing tissue malignancy would be expected. However, there is limited evidence to indicate how carbonate is distributed between sites, although each may have differing effects on the physicochemical properties of HAp [22, 27]. For example, A- and B-type carbonate substitution have complementary effects on the crystallographic ‘a’ and ‘c’ lattice parameters [28]. Previous work has also reported an association of larger crystal coherence length with increasing malignancy [14].

As crystallites precipitate, features of the local tissue chemistry may become ‘immortalised’ within the individual crystals that subsequently remain stable until such time as resorption occurs. Thus, identifying differences in the microstructure of HAp calcifications in breast tissue may provide insights into relationships between tissue pathology and microcalcification structure. Detailed characterisation of calcification microstructure therefore has the potential for diagnostic utility, particularly when distinguishing between the range of breast pathologies observed. This preliminary study aims to further investigate the differences in HAp crystals found in benign and pathological breast tissue by analysing crystallite size and non-uniform strain using X-ray crystallographic analysis.

## Materials and Methods

### Tissue Specimens

Formalin-fixed, paraffin-embedded (FFPE) core biopsy breast specimens from the Gloucestershire Hospitals NHS Foundation Trust diagnostic archive from 2012 (under approval from the Gloucestershire Local Research Ethics Committee) were randomly selected subject to the presence of calcifications in the histopathology report, and a classification of ‘B2—Benign’, ‘B5a—Ductal Carcinoma In Situ’, or ‘B5b—Invasive Carcinoma’ [14]. Blocks were provided with a histopathology report further detailing any associated necrosis and microanatomic location and subsequently scanned using a Nikon Metrology XT H225 CT system at 20 kV to select for significant levels of calcification. Where samples were classified as Benign, the histopathology reports fibrocystic changes, and in some cases, hyperplasia or fibroadenoma, with no evidence of malignancy. Calcification was associated with fibroadenoma or sclerotic stroma. Where samples were classified as Ductal Carcinoma in Situ, they exhibit intermediate or high grade DCIS pathology, there was evidence of comedo necrosis in 2/4 of samples and there was no evidence of invasive malignancy within the biopsies. Calcifications were located inside the breast duct and associated with comedo necrosis, where present. Where samples were classified as Invasive Carcinoma, there was associated DCIS in 4/6 cases, and only calcifications associated with the invasive component were measured.

### Crystallographic Analysis

5 μm sections were cut from each block and mounted on 12.5 μm thick polyolefin heat shrink films stretched over 38 mm diameter aluminium alloy rings and held in place with rubber ‘O’ rings. Geometric calibrations of the instrumentation were undertaken using a National Institute of Standards & Technology, Standard Reference Material 640c silicon powder. This was made into slurry in a dilute solution of PVA adhesive and painted onto the mounting films in the plane of the tissue sections [14].

Data collections were carried out at the Diamond Light Source, Didcot, UK on beamline i18. The sample rings were clamped perpendicular to the X-ray beam on a motorized stage and measurements made in transmission using a beam spot size of 10 × 10 μm, and an energy of 10.0 keV. Data was collected from 11 equally spaced positions in a vertical line across each calcification with an exposure time of 30 s per point. In total, 548 diffractograms were collected from 55 separate calcifications in 15 specimens, consisting of 5 benign, 4 in-situ, and 6 invasive (16, 17 and 22 calcifications respectively).

### Data Analysis

The 1-D data produced (“diffractograms”), exhibited a large background to signal ratio, and therefore the background was subtracted using Eva (BRUXER AXS) before microstructural analysis in TOPAS 4.2 software (BRUXER AXS). Crystallographic phase identification was carried out using the International Centre for Diffraction Data (ICDD) database (PDF-4, 2018).

A valuable feature of X-ray diffraction as an analytical approach is that CL may be determined for different crystallographic directions and thus provides access to several independent potential biomarkers [29, 30]. Each unique diffraction peak represents scattering from crystallographic planes in specific orientations and thus, by appropriate choice of Bragg maxima, the CL may be considered in three directions, <00ℓ>, <hk0> and < 0 k0>.

For each diffractogram, the HAp coherence length (CL) was calculated from the fitted full-width half-maximum (β) of the (002), (004), (030) and (210) diffraction maxima, using the Scherrer equation [30]:

1 $$ CL=\frac{K\lambda}{\beta \cos \kern0.3em \theta } $$

where K is the Scherrer constant (0.9), λ is the X-ray wavelength (0.124 nm) and θ is the Bragg angle.

The anisotropy of hydroxyapatite crystals means size and non-uniform strain must be separated for each crystallographic direction. Profile fitting of hk0 and 0 k0 peaks other than (210) and (030) are unreliable due to overlapping and therefore accurate separation of crystallite size and strain is not possible for the associated directions. Therefore, the CL was segregated into crystallite size and non-uniform strain calculated for the <00ℓ> direction from the (002) and (004) maxima using the Williamson-Hall method [31]:

2 $$ \beta \cos \theta =4\varepsilon \sin \theta +\frac{K\lambda}{L} $$

where β is the FWHM,, L is the crystallite size,, and ε is the non-uniform strain.

Parameterised data (coherence length, crystallite size and non-uniform strain) was averaged per calcification before carrying out statistical analyses. Data was tested for normality using the Anderson-Darling test at a 95% confidence level. Where data did not possess a normal distribution, statistical analysis between groups was carried out using the Mann-Whitney U test at a 95% confidence level in Minitab 17 Statistical Software.

Relative values of coherence length, crystallite size and non-uniform strain are compared below.

## Results

**Fig. 2:**
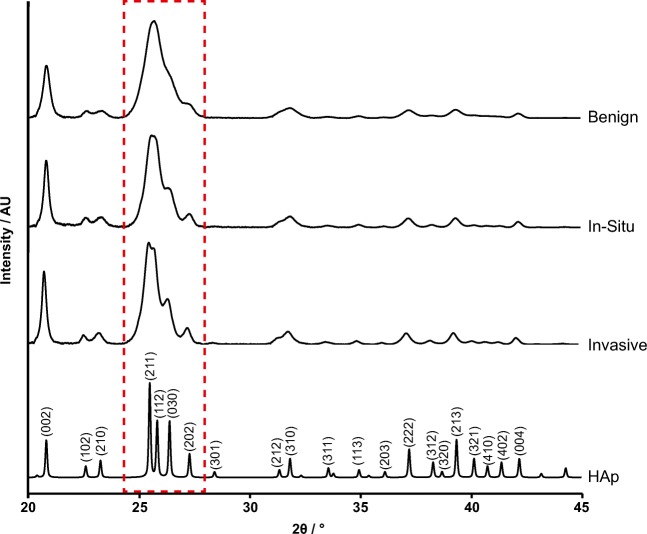
Normalised X-ray diffractograms of calcifications associated with benign, in-situ and invasive breast tissue and stochiometric HAp. A decrease in peak broadening from benign to invasive is highlighted between 24 and 29 ° (boxed), indicating a change in crystallinity

The averaged diffractograms of benign, in-situ and invasive calcifications and a stochiometric, highly crystalline HAp are shown in Fig. 2. Tissue calcifications in all three conditions were identified uniquely as HAp. Comparing diffractograms from the biogenic HAp to that of the stoichiometric sample, it is clear that the diffraction peaks are broadened in all three biogenic cases, indicating that none of these samples consist of structurally ordered HAp. This is typical of biogenic apatites such as bone mineral. Overall peak broadening (related to coherence length, see Eq. 1) decreased from benign to in-situ to invasive calcifications. This is particularly apparent in the overlapping group of 211, 112, 030 and 202 maxima which become progressively better defined with increasing malignancy.

**Fig. 3:**
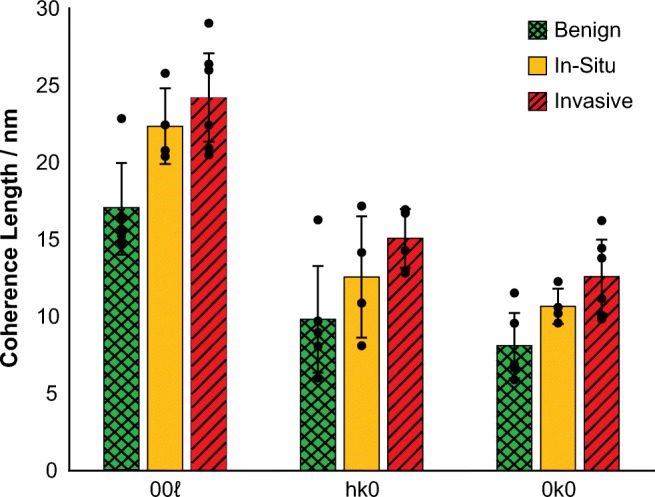
Coherence length measurements for benign, in-situ and invasive calcifications in the 00ℓ, hk0 and 0 k0 directions. Marked points indicate the average values for each sample. Statistical analysis was carried out using Mann-Whitney U test, **P < 0.01, ***P < 0.001. Error bars represent 95% confidence intervals

There was a significant increase in CL between benign and in-situ calcifications in <00ℓ> (*p* < 0.01) and < 0 k0> (*p* < 0.01) directions, and between in-situ and invasive for the <0 k0> direction (*p* < 0.01), as shown in Fig. 3. The relative difference between benign and in-situ calcifications for the <00ℓ>, <hk0> and < 0 k0> directions are similar (25%, 22% and 27%), while the difference between in-situ and invasive is smaller than the difference between benign and in-situ in all three cases. However, the percentage difference between in-situ and invasive calcifications is more pronounced in the <0 k0> and < hk0> directions (23% and 19%) compared to the <00ℓ> (11%) direction.

**Fig. 4:**
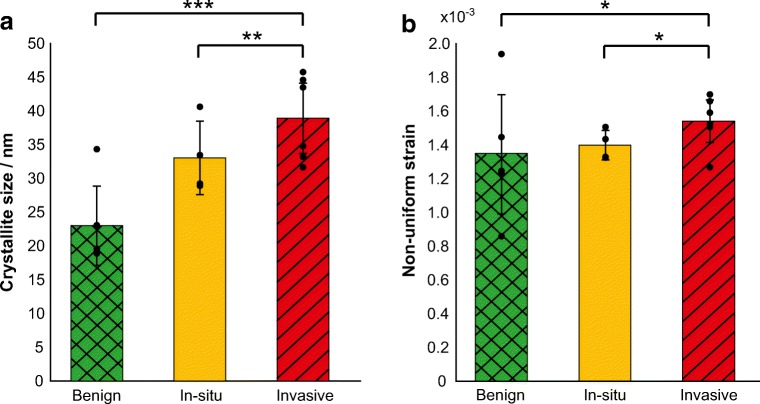
Crystallite size (**a**) and non-uniform strain (**b**) measurements for benign (*n* = 5), in-situ (*n* = 4) and invasive (*n* = 6) calcifications in the 00ℓ direction. Marked points indicate the average value for each sample. Statistical analysis was carried out using Mann-Whitney U test, *P < 0.05, **P < 0.01, ***P < 0.001. Error bars represent 95% confidence intervals

A significant increase in crystallite size with increasing malignancy (*p* < 0.05, Fig. 4a) was demonstrated. The crystallite size (in the <00ℓ> direction) increased by 40% from benign to in-situ tissues (*p* < 0.01), whereas an increase of 21% was observed from in-situ to invasive (*p* < 0.05). Non-uniform strain was also found to significantly increase with malignancy. Statistically significant differences were observed between benign and invasive (*p* < 0.05) and in-situ and invasive (*p* < 0.05) calcifications (Fig. 4b). In contrast to differences in coherence length and crystallite size, there was a greater increase from in-situ to invasive calcification non-uniform strain (42%) compared to benign to in-situ (7%).

## Discussion

**Fig. 5:**
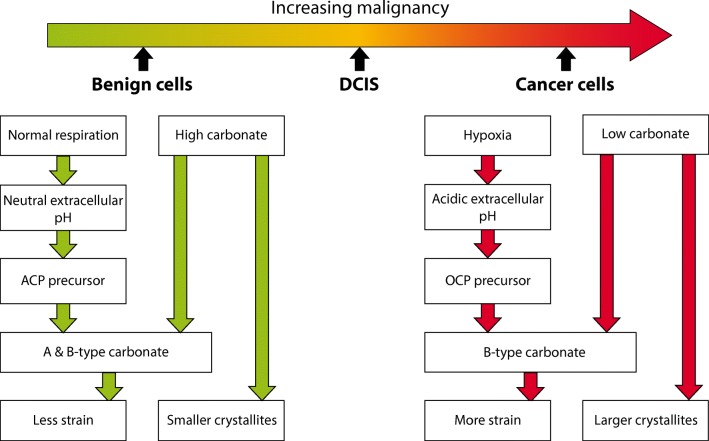
Summary diagram showing the microenvironmental factors affecting the incorporation of different types of carbonate into HAp in microcalcifications associated with benign, DCIS and invasive breast tissue cells

In summary, our findings demonstrate significant increases in coherence length, crystallite size and non-uniform strain of calcifications with increasing tissue malignancy. Values of these characteristics were comparable to other natural apatites; with size ranging from 23 to 39 nm and non-uniform strain in the order of 10^−3^ [21, 25]. These findings support the view that the mechanisms of calcification formation in different tissue microenvironments may vary, thus below we propose a new model for this process. This is summarised in Fig. 5.

### Tissue Microenvironment and Calcification Formation

Tissue microenvironments surrounding benign, in-situ and invasive cells can differ greatly and therefore the ionic milieu within which calcifications form is varied. The ability of an invasive cell to metastasise is governed by the conditions in its microenvironment, which is moderated by numerous factors including pH, proteins, the extracellular matrix, cellular processes and ion concentrations [12]. Biogenic calcifications are also known to possess different characteristics, such as phase composition and carbonate content when associated with different tissue pathologies [14, 15, 22].

Acidity is an important factor for calcification formation. Extracellular acidity is also a key marker for cancer aggressiveness, caused by the switch to a glycolytic respiration type, induced by hypoxia in tumour cells, known as the Warburg effect. Glycolysis produces a higher level of hydrogen ions compared to aerobic respiration, which in turn efflux from the cytoplasm to maintain a neutral or alkaline intracellular pH, causing the extracellular pH to become more acidic [12, 32, 33]. In addition, the action of carbonic anhydrases (CA), intracellularly and extracellularly, leads to an acidic extracellular pH due to the conversion of water carbon dioxide to hydrogen and bicarbonate ions in the extracellular fluid [34]. The expression of one extracellularly acting CA, CAIX, has also been associated with higher DCIS grade and invasive carcinoma, concurrent with an increasingly acidic extracellular pH with malignancy [35, 36].

An impact on calcification formation is reported in studies where an acidic pH increases the likelihood of forming an acidic precursor during the hydroxyapatite formation process [11]. Neutral and alkaline pH favours the direct conversion from ACP to HAp, whereas an acidic pH causes the formation of an additional precursor such as OCP [37]. Therefore, differences in extracellular pH between benign, in-situ and invasive cells would result in different formation mechanisms for hydroxyapatite microcalcifications. These different formation mechanisms lead to the incorporation of different types of carbonate substitution; ACP hydrolysis favouring substitution of carbonate into both the hydroxide site (A-type) and phosphate site (B-type) whereas OCP hydrolysis favours the incorporation of carbonate within the B-type sites [38]. Therefore, benign cell, neutral pH environments promote ACP hydrolysis that is more likely to produce A/B carbonated apatites, whereas invasive cell environments, (favouring OCP hydrolysis), would result in predominantly B-type carbonate apatites. This is further supported by the observations that sodium incorporation into the HAp lattice induces OCP formation, and sodium substitution is highest in invasive calcifications [15, 39]. Hypoxia has also been shown to initiate osteogenic differentiation in other cell types, therefore may contribute to the differing formation mechanisms between benign, in-situ and invasive breast tissue pathologies [40].

Additionally, at low carbonate weight percentages (<4 wt%), a lower carbonate wt% has been associated with B-type carbonate substitution, and a higher wt% favours A-type [20]. Taken together with studies reporting a lower total carbonate level in invasive calcifications and increasing levels with decreasing malignancy, this suggests the favouring of B-type carbonate in invasive calcifications, and A-type in benign [22].

Together the acidic extracellular pH and previously reported carbonate concentrations in microcalcifications suggests a higher level of B-type carbonate in HAp calcifications associated with invasive tissue pathology. Equally, the neutral pH and higher carbonate wt%of benign cells suggest an increased A-type carbonate incorporation. These mechanisms are reflected in the microstructure measurements and lattice parameters measured in benign, in-situ and invasive calcifications.

### Calcification Microstructure

Previous studies have demonstrated a decrease in total carbonate substitution with increasing tissue malignancy, which should lead to an increase in crystallite size with malignancy [17, 22]. This observation is also supported by our data; microcalcifications associated with invading tumours were characterised to possess the largest crystallite size (Fig. 4) For the first time, our analysis also enables a more specific inference regarding the lattice carbonate distribution.

Increasing total carbonate substitution has also been linked to an increase in non-uniform strain, therefore a decrease in non-uniform strain with malignancy would be expected [21]. However, the data presented here indicates the opposite trend. B-type substitutions have previously been shown to increase non-uniform strain [17]. B-type substitutions will, in principle, for the same number of substitutions, produce greater non-uniform strain than that of A-type. This is because carbonate substitution for phosphate occurs randomly at 6 crystallographically equivalent sites (*hm*) within the unit cell, compared to 2 (*e3*) for hydroxide substitutions. The non-uniform strain values observed within this study, suggests an increasing B-type carbonate substitution with increasing malignancy. However, given the total carbonate decreases with malignancy, this study suggests that lattice carbonate of breast microcalcifications occupies both A-type and B-type sites [22].

This model is further supported by previous estimates of apatite unit cell dimensions as revealed by lattice parameter measurements. A-type carbonate substitutions increase the apatite ‘a’ axis due to CO_3_ having a significantly greater ionic radius than OH. In contrast, B-type carbonate decreases the ‘a’ axis due to CO_3_ having a smaller radius than phosphate [28]. In addition, A-type carbonate substitutions decreases the ‘c’ axis and B-type increases the ‘c’ axis [28]. Previous studies have shown a decrease in the ‘a’ axis with malignancy and an increase in the ‘c’ axis, further supporting the view that the quantity of B-type carbonate substitution increases and A-type substitution decreases with increasing malignancy [14].

The distribution of data observed in all the Williamson-Hall plots is indicative of microstructural anisotropy. The lack of reliable peaks in reflections other than the 00ℓ means that only data in this reflection could be separated into size and non-uniform strain. However, the noted increases in CL with malignancy in all three reflections analysed (Fig. 3) suggest that crystallite size and/or non-uniform strain are also changing in the <hk0> and < 0 k0> directions. Proteins have been noted to interact with the non-basal faces of HAp, restricting growth in neutral pH [41]. Extracellular acidity may cause changes in protein conformation in cancer cells permitting increased CL in the <0 k0> and < hk0> directions, hence a directional variance in difference magnitude. Thus, for the development of quantitative biomarkers based upon microstructural features, appropriate choice of lattice direction to maximise sensitivity is required.

### The Importance of Microstructural Differences

Studies have previously shown that the total carbonate content of hydroxyapatite is positively correlated with the solubility of the crystals, which would suggest benign breast calcifications are more soluble than pathological ones as a consequence of their respective carbonate contents [21, 22]. However, more stable calcifications are generally considered to be benign [42]. A positive correlation between non-uniform strain and carbonated apatite solubility has previously been found, which may offer an explanation for this apparent contradiction, where less strained crystallites, i.e. those in benign calcifications, are less soluble [21]. This may suggest that the type of carbonate also has a role to play in other calcification characteristics. In addition, a more acidic pH increases the solubility of hydroxyapatite, which may also play a role in the relative instability of calcifications in pathological tissue [43].

Furthermore, the interaction of calcification with proteins in the surrounding tissue has been shown to play an important role in the propagation of tumour virulence characteristics. For example, the presence of hydroxyapatite in mammary cell lines has been shown to upregulate matrix metalloproteinases which are key proteins in the degradation of the basement membrane, enhance mitogenesis, and induce the production of interleukins (ILs) [44]. It has also been demonstrated that the surface morphology of hydroxyapatite crystals impacts the level of protein adsorption on to the crystals, which can have downstream effects on expression of osteoblastic markers such as Runt-related transcription factor 2 (RUNX2) and alkaline phosphatase (ALP) [45]. Moreover, the substitution of ions such as carbonate into the hydroxyapatite lattice can affect crystal morphology, meaning that the microstructure of microcalcifications potentially has the ability to impact downstream effectors and hence tumour virulence characteristics [20, 46].

## Conclusions

This paper presents a preliminary set of data that indicates a role for X-ray diffraction in understanding breast microcalcification chemistry and formation. The data presented in this paper demonstrates, firstly, that there are measurable differences in the microstructure of hydroxyapatite calcifications associated with benign, in-situ and invasive breast tissue pathologies. Secondly, the data suggests that a combination of A-type and B-type substituted carbonate is found in breast microcalcifications, and it is the ratio of these two types that is specifically related to tissue type. Finally, the differing tissue microenvironments, caused by differing tissue metabolisms, combined with the model presented here indicate that the tissue pathology governs the formation mechanisms and therefore the composition of calcifications, meaning tissue pathology is immortalised within microcalcifications microstructure in the breast tissue. These differences may have a multitude of impacts on the propagation of tumour characteristics through interactions with the microenvironment. We appreciate that the model proposed is based upon limited observations and therefore remains somewhat speculative. In addition, it is not possible to determine whether invasive calcifications are ‘left-over’ from previous DCIS, but this may be an avenue to explore in future research. Using an expanded data set, this model can be developed into a defined algorithm, which may cement microstructural parameters as a key prognostic indicator for breast disease in the future.

## Data Availability

The datasets generated during and/or analysed during the current study are available from the corresponding author on reasonable request.

## References

[1] Office for National Statistics. Cancer Registration Statistics, England 2016 [Internet]. Cancer Regist. Stat. Engl. 2016. 2018.

[2] Rayat P. Breast screening Programme, England, 2016-17 [internet]. NHS Digit 2018.

[3] Sickles EA, D-Orsi CJ, Bassett LW. ACR BI-RADS Atlas, Breast Imaging Reporting and Data System. Am Coll Radiol. 2013.

[4] Henrot P, Leroux A, Barlier C, Génin P (2014). Breast microcalcifications: the lesions in anatomical pathology. Diagn Interv Imaging Elsevier Masson SAS.

[5] Stomper PC, Geradts J, Edge SB, Levine EG. Mammographic predictors of the lesions without a mass. Medicine (Baltimore). 2003:1679–84. 10.2214/ajr.181.6.1811679 14627596

[6] Frappart L, Boudeulle M, Boumendil J, Lin HC, Martinon I, Palayer C, Mallet-Guy Y, Raudrant D, Bremond A, Rochet Y (1984). Structure and composition of microcalcifications in benign and malignant lesions of the breast: study by light microscopy, transmission and scanning electron microscopy, microprobe analysis, and X-ray diffraction. Hum Pathol.

[7] Scimeca M, Bonfiglio R, Montanaro M, Bonanno E (2018). Osteoblast-like cells in human cancers: new cell type and reliable markers for bone metastasis. Future Oncol.

[8] Bellahcène A, Castronovo V (1995). Increased expression of osteonectin and osteopontin, two bone matrix proteins, in human breast cancer. Am J Pathol.

[9] Vidavsky N, Kunitake JA, Chiou AE, Northrup PA, Porri TJ, Ling L, Fischbach C, Estroff LA (2018). Studying biomineralization pathways in a 3D culture model of breast cancer microcalcifications. Biomaterials..

[10] Guinebretière JM, Menet E, Tardivon A, Cherel P, Vanel D (2005). Normal and pathological breast, the histological basis. Eur J Radiol.

[11] Wang L, Nancollas GH (2008). Calcium orthophosphates: crystallization and dissolution. Chem Rev.

[12] Damaghi M, Wojtkowiak JW, Gillies RJ (2013). pH sensing and regulation in cancer. Front Physiol.

[13] Place AE, Jin Huh S, Polyak K (2011). The microenvironment in breast cancer progression: biology and implications for treatment. Breast Cancer Res.

[14] Scott R, Stone N, Kendall C, Geraki K, Rogers K (2016). Relationships between pathology and crystal structure in breast calcifications: an in situ X-ray diffraction study in histological sections npj. Breast Cancer.

[15] Scott R, Kendall C, Stone N, Rogers K (2017). Elemental vs phase composition of breast calcifications. Sci Rep.

[16] Combes C, Cazalbou S, Rey C. Apatite Biominerals. Minerals. 2016.

[17] Venkateswarlu K, Sandhyarani M, Nellaippan T, Rameshbabu N (2014). Estimation of crystallite size, lattice strain and dislocation density of nanocrystalline carbonate substituted hydroxyapatite by X-ray peak variance analysis. Procedia Mater Sci.

[18] LeGeros RZ, Apatites IN (1981). Biological systems. Prog Cryst Growth Charact.

[19] Hargreaves JSJ (2016). Some considerations related to the use of the Scherrer equation in powder X-ray diffraction as applied to heterogeneous catalysts some considerations related to the use of the Scherrer equation in powder X-ray diffraction as applied to heterogeneous catal. Catal Struct React. Taylor & Francis.

[20] Barralet J, Best S, Bonfield W (1998). Carbonate substitution in precipitated hydroxyapatite: an investigation into the effects of reaction temperature and bicarbonate ion concentration. J Biomed Mater Res.

[21] Baig AA, Fox JL, Young RA, Wang Z, Hsu J, Higuchi WI, Chhettry A, Zhuang H, Otsuka M (1999). Relationships among carbonated apatite solubility, crystallite size, and microstrain parameters. Calcif Tissue Int.

[22] Baker R, Rogers KD, Shepherd N, Stone N (2010). New relationships between breast microcalcifications and cancer. Br J Cancer. Nature Publishing Group.

[23] Yusufoglu Y, Akinc M (2007). Effect of pH on the carbonate incorporation into the hydroxyapatite prepared by an oxidative decomposition of calcium-EDTA chelate. J Am Ceram Soc.

[24] Rey C, Collins B, Goehl T, Dickson IR, Glimcher MJ (1989). The carbonate environment in bone mineral: a resolution-enhanced fourier transform infrared spectroscopy study. Calcif Tissue Int.

[25] Ortiz-Ruiz AJ, Teruel-Fernández J d D, Alcolea-Rubio LA, Hernández-Fernández A, Martínez-Beneyto Y, Gispert-Guirado F (2018). Structural differences in enamel and dentin in human, bovine, porcine, and ovine teeth. Ann Anat.

[26] Baker RN, Rogers KD, Shepherd N, Stone N, Schweitzer D, Fitzmaurice M (2007). Analysis of breast tissue calcifications using FTIR spectroscopy. Proc SPIE-Int Soc Opt Eng.

[27] Haka AS, Shafer-Peltier KE, Fitzmaurice M, Crowe J, Dasari RR, Feld MS (2002). Identifying microcalcifications in benign and malignant breast lesions by probing differences in their chemical composition using Raman spectroscopy. Cancer Res.

[28] LeGeros RZ, Trautz OR, Klein E, LeGeros JP (1969). Two types of carbonate substitution in the apatite structure. Experientia..

[29] Greenwood C, Rogers K, Beckett S, Clement J (2013). Initial observations of dynamically heated bone. Cryst Res Technol.

[30] Scherrer P (1918). Bestimmung der Größe und der inneren Struktur von Kolloidteilchen mittels Röntgenstrahlen. Nachrichten von der Gesellschaft der Wissenschaften zu Göttingen, Math Klasse.

[31] Williamson G, Hall W (1953). X-ray line broadening from filed aluminium and wolfram. Acta Metall.

[32] Gatenby RA, Gillies RJ (2004). Why do cancers have high aerobic glycolysis?. Nat Rev Cancer.

[33] Lagadic-Gossmann D, Huc L, Lecureur V (2004). Alterations of intracellular pH homeostasis in apoptosis: origins and roles. Cell Death Differ.

[34] Tafreshi NK, Lloyd MC, Proemsey JB, Bui MM, Kim J, Gillies RJ, Morse DL (2016). Evaluation of CAIX and CAXII expression in breast Cancer at varied O 2 levels: CAIX is the superior surrogate imaging biomarker of tumor hypoxia HHS public access. Mol Imaging Biol.

[35] Wykoff CC, Beasley N, Watson PH, Campo L, Chia SK, English R, Pastorek J, Sly WS, Ratcliffe P, Harris AL (2001). Expression of the Hypoxia-Inducible and Tumor- Associated Carbonic Anhydrases in Ductal Carcinoma in Situ of the Breast. Am J Pathol American Society for Investigative Pathology.

[36] Lobo RC, Hubbard NE, Damonte P, Mori H, Pénzváltó Z, Pham C, et al. Glucose uptake and intracellular pH in a mouse model of ductal carcinoma in situ ( DCIS ) suggests metabolic heterogeneity. 2016;4:1–10. 10.3389/fcell.2016.00093 PMC5005977 27630987

[37] Johnsson MS-A, Nancollas GH (1992). The role of brushite and octacalcium phosphate in apatite formation. Crit Rev Oral Biol Med.

[38] Tomazic BB, Mayer I, Brown WE (1991). Ion incorporation into octacalcium phosphate hydrolyzates. J Cryst Growth.

[39] Sugiura Y, Makita Y (2018). Sodium induces octacalcium phosphate formation and enhances its layer structure by affecting the hydrous layer phosphate. Cryst Growth Des.

[40] Balogh E, Tóth A, Méhes G, Trencsényi G, Paragh G, Jeney V. Hypoxia triggers osteochondrogenic differentiation of vascular smooth muscle cells in an HIF-1 ( Hypoxia- Inducible Factor 1 )– dependent and reactive oxygen species – dependent manner. 2019;1:1088–99. 10.1161/ATVBAHA.119.312509 31070451

[41] Xu Zhijun, Yang Yang, Wang Ziqiu, Mkhonto Donald, Shang Cheng, Liu Zhi-Pan, Cui Qiang, Sahai Nita (2013). Small molecule-mediated control of hydroxyapatite growth: Free energy calculations benchmarked to density functional theory. Journal of Computational Chemistry.

[42] Lev-Toaff AS, Feig SA, Saitas VL, Finkel GC, Schwartz GF (1994). Stability of malignant breast microcalcifications. Radiology.

[43] Pan H, Darvell BW (2010). Effect of carbonate on hydroxyapatite solubility. Cryst Growth Des.

[44] Morgan Maria P., Cooke Michelle M., McCarthy Geraldine M. (2005). Microcalcifications Associated with Breast Cancer: An Epiphenomenon or Biologically Significant Feature of Selected Tumors?. Journal of Mammary Gland Biology and Neoplasia.

[45] Yang W, Han W, He W, Li J, Wang J, Feng H (2016). Surface topography of hydroxyapatite promotes osteogenic differentiation of human bone marrow mesenchymal stem cells. Mater Sci Eng C. Elsevier B.V..

[46] Liao S, Watari F, Xu G, Ngiam M, Ramakrishna S, Chan CK (2007). Morphological effects of variant carbonates in biomimetic hydroxyapatite. Mater Lett.

